# From Myofascial Chains to the Polyconnective Network: A Novel Approach to Biomechanics and Rehabilitation Based on Graph Theory

**DOI:** 10.3390/life15081200

**Published:** 2025-07-28

**Authors:** Daniele Della Posta, Immacolata Belviso, Jacopo Junio Valerio Branca, Ferdinando Paternostro, Carla Stecco

**Affiliations:** 1 The Anatomical Network APS, Via Fermo 2c, 00182 Rome, Italy; osteodan@gmail.com; 2 Department of Psychology and Health Sciences, Telematic University Pegaso, 80143 Naples, Italy; 3 Department of Experimental and Clinical Medicine, University of Florence, 50134 Florence, Italy; ferdinando.paternostro@unifi.it; 4 Department of Neuroscience, Institute of Human Anatomy, University of Padova, 35141 Padova, Italy; carla.stecco@unipd.it

**Keywords:** anatomical network, myofascial network, osteo-myofascial system, functional anatomy, biomechanical modeling

## Abstract

In recent years, the concept of the myofascial network has transformed biomechanical understanding by emphasizing the body as an integrated, multidirectional system. This study advances that paradigm by applying graph theory to model the osteo-myofascial system as an anatomical network, enabling the identification of topologically central nodes involved in force transmission, stability, and coordination. Using the aNETomy model and the BIOMECH 3.4 database, we constructed an undirected network of 2208 anatomical nodes and 7377 biomechanical relationships. Centrality analysis (degree, betweenness, and closeness) revealed that structures such as the sacrum and thoracolumbar fascia exhibit high connectivity and strategic importance within the network. These findings, while derived from a theoretical modeling approach, suggest that such key nodes may inform targeted treatment strategies, particularly in complex or compensatory musculoskeletal conditions. The proposed concept of a polyconnective skeleton (PCS) synthesizes the most influential anatomical hubs into a functional core of the system. This framework may support future clinical and technological applications, including integration with imaging modalities, real-time monitoring, and predictive modeling for personalized and preventive medicine.

## 1. Introduction

Throughout the 20th century, several authors from different disciplines, such as physiotherapy, osteopathy and manual medicine, have contributed to the evolving concept of the myofascial chains, emphasizing the body’s interconnection [1,2,3,4,5,6]. While this model offers a more integrated view of musculoskeletal dynamics, it often relies on linear or unidirectional pathways [1,2,3,4,5,6]. This introduces significant limitations, as it tends to describe force distribution in a rigid and unidimensional manner. Growing awareness of these limitations has driven researchers to explore alternative models that better represent the complexity and multi-directionality of the osteo-myofascial network. Despite their conceptual value, linear chain models fall short in capturing the multidirectional and adaptive behavior of the osteo-myofascial system. They tend to over-simplify force distribution and structural integration, prompting the need for more sophisticated representations. Among these, graph analysis, as proposed by the authors in previous works, offers new perspectives. Through a three-dimensional network model of the osteo-myofascial system, called aNETomy, it is now possible not only to visualize the system’s non-linearity, but also to analyze its connections, revealing functional aspects that determine its adaptive flexibility in response to postural and traumatic stress. This methodology also offers novel clinical potential for diagnosis, treatment, and personalized therapeutic protocols. This study aims to model the osteo-myofascial system as an anatomical network using graph theory, and to identify high-centrality nodes that may serve as key targets for biomechanical analysis and therapeutic intervention.

## 2. Materials and Methods

The evolution from a linear model to a three-dimensional network paradigm marks a crucial shift in our understanding of human muscular anatomy, with implications for both the morphological evolution and the functional organization of the locomotor system [7,8,9]. The in silico representation of the osteo-myofascial system allows for the study of this complexity, making structural non-linearity explicit and enabling a mathematical analysis of anatomical relationships in their entirety. This approach paves the way for a genuinely holistic understanding of the osteo-myofascial structure. Unlike linear models, the anatomical network enables the visualization of the entire body as a complex web of nodes and edges, where each anatomical structure (muscles, bones, and fascial compartments) acts as a pivotal point within an interconnected and multidirectional system. Each element holds a different weight based on topological and functional attributes [10]. Although the relationships between anatomical structures in an anatomical network are inherently static (i.e., they vary negligibly between individuals), they are still non-linear and they exhibit functional variability. This is due to the multidimensional and viscoelastic properties of the tissues involved, allowing the body to distribute and absorb forces dynamically across multiple paths. The presence of an extremely high number of potential force-transmission paths has been computed [11], indicating that each individual possesses a unique configuration of nodes and connections influenced by posture, injury history, and motor habits.

### 2.1. Network Model Description

This study employed the aNETomy anatomical network model (The Anatomical Network, available at http://anetomy.net/). aNETomy enables the construction of anatomical networks based on relational databases, producing an undirected graph in which each node represents a specific anatomical structure (e.g., muscle, bone, and fascial compartments) and each edge represents a functional or contiguous relationship between structures. The osteo-myofascial (or biomechanical) graph was created and validated using anatomical data [12] describing fascial and muscular connections within the human body. The network was visualized using Cytoscape [13] and Gephi [14], two software tools used for modeling biological and complex systems. This model enables the exploration of the network as a 3D, multidirectional system, shedding light on how anatomical nodes are functionally organized to ensure stability and efficiency in musculoskeletal dynamics. Furthermore, the model allows the human body to be interpreted as a set of adaptive and flexible pathways, capable of responding to structural and functional stressors. This reflects the scalability and complexity of real-world networks, which evolve in response to changing conditions [15,16,17].

### 2.2. Data Source

Data on the osteo-myofascial network were extracted from the BIOMECH 3.4 relational database, structured in tables describing nodes (anatomical structures) and connections (relationships between them), verified via anatomical atlases and dissection [12].

### 2.3. Network Construction

Data extraction was performed using SQL queries, obtaining

- A list of nodes with unique identifiers and descriptions;
- A list of connections as node pairs (source and target), with optional weights indicating relationship strength.

The data were imported into Python and processed using the NetworkX library (NetworkX Documentation, available at https://networkx.org) to

- Construct the graph;
- Calculate degree, betweenness, and closeness centralities;
- Generate the adjacency matrix for structural analysis.

The results were visualized in Cytoscape [13] and Gephi [14] to confirm topological properties and identify key anatomical hubs. Given the statistical nature of the anatomical data, the resulting network is a topological representation that reflects structural relationships rather than real-time dynamics. Therefore, interpretation must be contextualized within the limitation of a static modeling, such as the lack of a viscoelastic variability or physiological adaptation.

### 2.4. Centrality Metrics Applied

**Figure 1:**
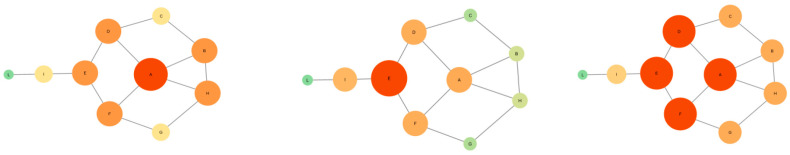
The image shows three network graphs highlighting different centrality measures: degree centrality, betweenness centrality, and closeness centrality. Letters A–M are only indicative labels used to distinguish nodes within the sample network and do not correspond to specific anatomical structures.

We selected degrees, betweenness, and closeness centrality due to their complementary roles in characterizing network structure. Degree centrality highlights local connectivity, betweenness identifies nodes acting as bridges, and closeness captures the efficiency of global communication (Figure 1).

Network analysis was performed using topological metrics to determine the role and importance of each node within the polyconnective network [18]. Three key metrics were selected for this study, each offering complementary insights into the anatomical–functional architecture of the system.

- Degree Centrality

This metric measures the number of direct connections a node has.

Mathematically,

$$C_{D}\left(v\right)=\frac{d\left(v\right)}{n-1}$$

where

*d*(*v*) is the number of nodes directly connected to node *v* (its degree);

*n* is the total number of nodes in the network.

The term *n* − 1 represents the maximum possible degree (excluding the node itself).

Nodes with high degree centrality play a locally influential role in force distribution within circumscribed regions.

- Betweenness Centrality

This metric assesses how often a node acts as a bridge in the shortest paths between other nodes

(1) $$C_{B}\left(v\right)=\sum_{s\neq v\neq t}\frac{\sigma_{st}\left(v\right)}{\sigma_{st}}$$

where

*s* and *t* are two distinct nodes in the network (*s* ≠ *t* ≠ *v*);

*σ_st_* is the total number of shortest paths between *s* and *t*;

*σ_st_*(*v*) is the number of those paths that pass-through node *v*.

The summation considers all possible pairs of nodes *s* and *t* in the network.

Nodes with high betweenness centrality are crucial for integrating distant regions, maintaining continuity of movement, and preventing dysfunction.

- Closeness Centrality

This metric evaluates the proximity of a node to all other nodes in the network

$$C_{C}\left(v\right)=\frac{n-1}{\sum_{u\neq v}d\left(v,u\right)}$$

where

*d*(*v*, *u*) represents the distance (number of edges or path weight) between node *v* and node *u*, and *n* is the total number of nodes in the network. The summation ∑*_u_*_≠*v*_ *d*(*v*, *u*) indicates the total distance from node *v* to all other nodes. Nodes with high closeness centrality can rapidly transmit information or tension, supporting global coordination.

## 3. Results

The analysis of centrality within the biomechanical network represents one of the most advanced tools available for understanding the role of each node in the distribution of tension and functional stability of the human body [19].

Although the osteo-myofascial anatomical network represents a “static” system, due to the relatively consistent morphological conformation across the human population, natural anatomical variations were evaluated to determine their impact on the distribution of centrality measures. The analysis confirmed no statistically significant variations, validating the reliability and coherence of the centrality-based network approach [20]. As the model represents a static anatomical network derived from a single curated source, variance metrics are not directly applicable. However, future extensions incorporating interindividual anatomical variability could enable robust statistical analysis of the network topologies.

A key distinction must be made between static and dynamic networks (Table 1): static models are suitable for analyzing persistent, stable structures, while dynamic models are essential to understand systems that evolve over time [21].

**Figure 2:**
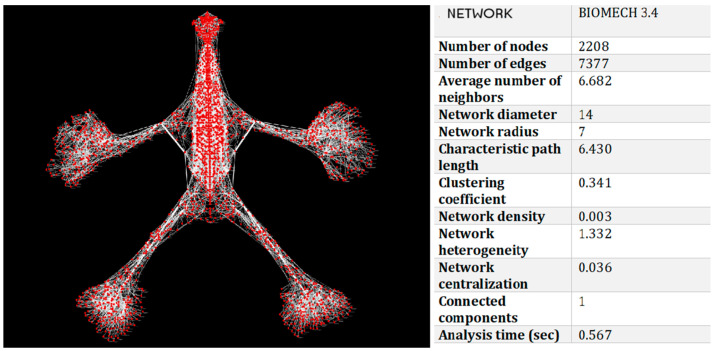
Representation of the osteo-myofascial network (BIOMECH 3.4), highlighting the complexity of the connections between anatomical structures and the integrated distribution of biomechanical interactions. Network analysis confirms a highly connected and antifragile configuration, with local functional clusters and systemic continuity across the entire osteo-myofascial network.

The network derived from the BIOMECH 3.4 model (Figure 2, Table 2) revealed a highly complex and integrated structure, consisting of

- 2208 nodes (anatomical structures);
- 7377 edges (biomechanical relationships).

**Figure 3:**
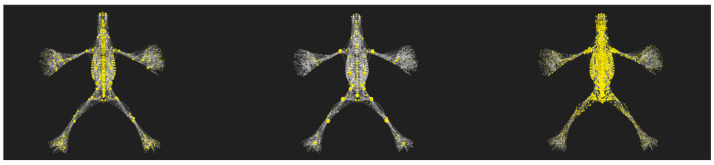
From left to right, the image shows the distribution of degree centrality, betweenness centrality, and closeness centrality within the aNETomy anatomical network model.

The graph theory framework enables the measurement of each node’s “centrality” in terms of its position and connectivity within the network. Among the various possible metrics, three centrality types emerged as clinically relevant (Figure 3).

### 3.1. Degree Centrality

Nodes with high degree centrality are highly locally connected, often acting as anatomical hubs for immediate force distribution. Structures such as the sternum, vertebrae, and sacrum serve as connective junctions, enhancing regional and global stability [8].

Clinical implication: Targeting these nodes in therapy may

- Improve local mobility;
- Release regional tension;
- Strengthen adjacent structural integrity [22].

### 3.2. Betweenness Centrality

Nodes with high betweenness centrality serve as bridges between distant regions, ensuring the continuity of movement and the modular integration of anatomical segments. Examples of these include

- The humerus;
- The iliotibial tract (fascia lata).

These structures channel forces between otherwise disconnected segments, reducing overload and enhancing coordination [11,20].

Therapeutic strategies focused on these “bridge” nodes may

- Reduce compensatory postures;
- Optimize load transfer;
- Enhance functional integration [23].

### 3.3. Closeness Centrality

This metric identifies nodes with high systemic accessibility: those capable of transmitting tension and signals rapidly and efficiently across the network. Typical examples of these include

- Lumbar vertebrae;
- The sacroiliac region.

These structures often mediate between hubs, synchronizing global motor responses (Table 3). Interventions on high-closeness nodes can

- Improve coordination;
- Enhance movement fluidity;
- Promote balanced force distribution [11,24].

A loss of function in these nodes may initiate cascading compensation, overload other hubs, and compromise the network’s adaptive capacity (Table 4).

### 3.4. Interpretation of Results and Clinical Considerations

The introduction of graph theory into the biomechanical and clinical fields offers a powerful tool for understanding and intervening in the polyconnective network in a precise and effective manner [23]. The PCS was derived by selecting nodes that scored in the upper percentile across all three centrality metrics. This subset highlights the most structurally and functionally influential anatomical structures, forming a functional core of the osteo-myofascial network. In this model, each anatomical structure is represented as a node within a complex network of connections, where the distribution of forces and postural balance depend on the spatial arrangement and dynamic behavior of the nodes themselves. Graph analysis allows for the identification of key structures, such as muscles or other relevant anatomical components, that act as central hubs for force transmission and load distribution, especially during daily or traumatic physical demands [24]. Centrality measures, such as closeness and betweenness, enable the recognition of those nodes that play a critical role in facilitating the transmission of mechanical tension throughout the musculoskeletal system. If compromised, this transmission could lead to functional congestion in certain areas, manifesting clinically as transient dysfunctions or chronic inflammatory–degenerative conditions [13]. In essence, identifying anatomical centralities translates clinically into the ability to target strategic areas for therapeutic interventions aimed at improving global functionality, reducing compensatory patterns or overload, and preventing injuries or degenerative locomotor disorders [22]. For example, a node with high betweenness centrality, capable of transmitting forces that influence multiple muscular or connective regions from periphery to center and vice versa, plays a pivotal role in channeling biomechanical forces from palmar, plantar, and facial peripheral structures, which are known to be crucial contact points for survival and relational life. Dysfunction in these centers, which are primarily located along the pathways of the upper and lower limbs, as well as the vertebral axis represented by the spinal dura mater (all of which converge at the sacral region), can disrupt the entire network. A dysfunction in this structure, referred to as the polyconnective skeleton, due to its role in integrating the various modules of the osteo-myofascial network, may generate perturbations that extend beyond the affected area. These perturbations can alter force and load distributions across adjacent modules, and through a process referred to as percolation, progressively affecting increasingly distant regions over time [25]. A patient with chronic pain caused by muscular or fascial tension may thus benefit from targeted interventions on high-betweenness nodes that form the polyconnective skeleton, helping to reduce diffuse tension and minimize postural compensations. Applying specific treatments to these key points of the polyconnective system can therefore accelerate recovery and prevent further injury [13]. Several central nodes identified (i.e., the sacrum, thoracolumbar fascia) confirm previous theoretical assumptions regarding biomechanical importance. However, the net-work-based identification of the PCS as a functionally integrated subset represents a novel contribution that formalizes and quantifies this concept through graph theory. On the other hand, it is important to underline that these clinical applications are currently inferred from topological data and have not yet been empirically validated in controlled studies. They should be interpreted as theoretical hypotheses based on anatomical modeling.

## 4. Discussion

Network analysis applied to the osteo-myofascial system offers an innovative and three-dimensional view of body biomechanics, overcoming the limitations of traditional linear models. Centrality metrics (degree, betweenness, and closeness) have made it possible to identify key nodes within the network, revealing structures that play essential roles in the transmission of forces, stability, and global coordination. The combined application of these metrics led to the definition of the polyconnective skeleton (PCS), an integrated structure that synthesizes the core functional roles of the biomechanical system. This subdivision into the full network, the residual network without the PCS, and the PCS alone, are shown in Figure 1, highlighting the differences in topological organization and centrality between configurations.

**Figure 4:**
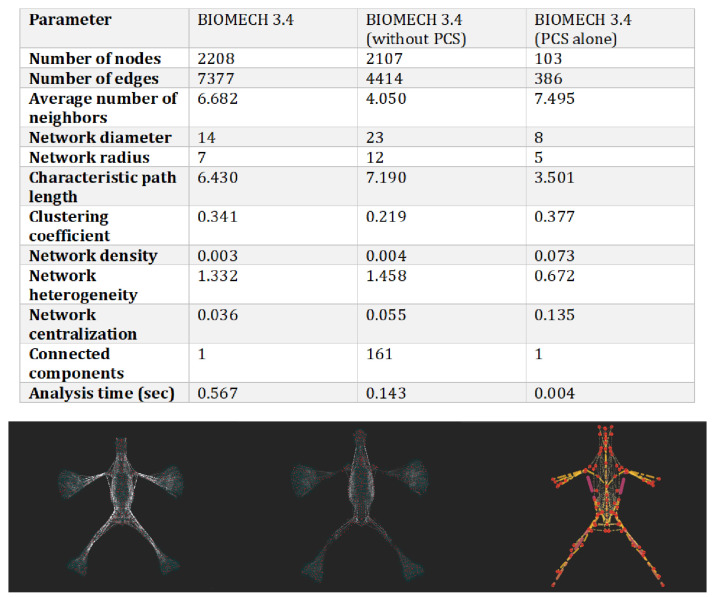
Comparison between the full network (BIOMECH 3.4), the residual network without the PCS (BIOMECH 3.4(6)), and the PCS alone (Merged Network_2).

The PCS emerges as a subset of high-centrality nodes that act as functional hubs within the osteo-myofascial network (Figure 4). Among them, the sacrum, thoracolumbar fasciae, and spinal dura mater perform complementary roles: nodes with high degree centrality ensure local stability and redistribute forces; those with high betweenness facilitate integration between distant segments, preventing rigidity and compensations; and those with high closeness allow for rapid tension transmission, optimizing systemic coordination.

The importance of the PCS goes beyond its structural description: it represents a functional framework capable of standardizing the interpretation of the biomechanical network. Thanks to its organization, the PCS provides a basis for analyzing complex functional aspects in a coherent way, identifying critical nodes that may influence the global balance and adaptive capabilities of the system. However, this framework is not rigid: through the analysis of a patient’s individual characteristics, the PCS can be used to personalize clinical interventions and create tailored therapeutic strategies.

From a clinical perspective, the standardization provided by the PCS makes it possible to identify recurring patterns of overload or dysfunction, which can be translated into targeted interventions. Treating central nodes, such as lumbar vertebrae and thoracolumbar fasciae, becomes essential for improving overall stability and coordination. Identifying specific nodes within the patient’s network context allows for interventions to be adapted to individual needs, reducing dysfunctional compensations and increasing the overall effectiveness of therapies. The sacrum and thoracolumbar fascia, identified as high-centrality nodes in the PCS, are common targets in manual therapies for lower-back pain. Interventions on the sacroiliac joint and thoracolumbar fascia have been associated with reduced pain and improved mobility, likely due to their key role in force transmission and postural integration [25]. These findings support the use of the PCS framework to guide therapeutic prioritization in cases with complex compensatory patterns.

Looking ahead, the PCS also represents an ideal platform for developing predictive tools based on network analysis. Integrating this model with advanced computational simulations and technologies such as dynamic imaging could allow for simulations of the network’s behavior under various loading conditions, predicting dysfunctions and identifying the most vulnerable nodes. This would not only improve diagnostic capacity, but also offer a preventive strategy to reduce the risk of injuries or chronic disorders.

In summary, the polyconnective skeleton represents the most significant outcome of centrality analysis applied to the osteo-myofascial system. It provides not only a structural and functional understanding of the network, but also opens new possibilities for personalized and predictive medicine, promoting an integrated and dynamic approach to managing the biomechanical system. Future research should focus on validating the PCS framework trough clinical correlations and enhancing it with real-time imaging, patient-specific modeling, and machine learning to support predictive and personalized rehabilitation strategies.

## 5. Conclusions

The application of graph theory to the polyconnective network allows for an in-depth analysis of the structural and functional organization of the osteo-myofascial system, highlighting the specific role of key nodes. This methodology provides insight into how these nodes contribute to force transmission, global stability, and tension regulation, offering a clearer understanding of the dynamics that govern biomechanical efficiency and adaptability. Moreover, analyzing the behavior of nodes that constitute the polyconnective skeleton (PCS) opens new possibilities for predictive diagnostics in biomechanical systems. This makes it feasible to identify potential dysfunctions before clinical symptoms emerge, an aspect of growing importance in elite sports medicine and injury prevention. For example, in elite sports medicine, the PCS could support predictive diagnostics by identifying anatomical hubs under repetitive stress. Indeed, multimodal data can be mapped on the PCS and processed by machine learning to anticipate dysfunction and adjust preventive strategies to the specific athlete’s profile.

In addition to topological analysis, integrating network metrics with instrumental data, such as myotonometry, electromyography, plantar pressure, fMRI, and dynamic ultrasound, can enhance the accuracy of clinical assessments. Together, these approaches support the development of personalized therapeutic protocols and preventive strategies based on a system-oriented understanding of the musculoskeletal network. Diagnostic imaging, such as dynamic ultrasound and fMRI, could support the real-time assessment of fascial behavior and functional connectivity in high-centrality nodes. However, the technical limitations (e.g., imaging depth, protocol variability, and anatomical standardization) currently hinder their routine clinical integration. Finally, the use of biomechanical computational models could further enhance our ability to simulate network behavior under different load and stress conditions, offering new insights for optimizing therapeutic interventions. The combination of these tools with network analysis would further strengthen the foundation for a comprehensive and predictive understanding of the polyconnective system, enhancing precision in overload pattern detection and improving clinical strategy development. In summary, graph-theoretical osteo-myofascial system modeling highlights the PCS as a central functional core. Although based on a static anatomical network, the PCS offers a promising foundation for predictive and personalized strategies, particularly when combined with functional data and imaging tools. The direction of current research not only strengthens the scientific validity of existing network models but also expands the potential for customizing treatment plans, contributing to more effective management of musculoskeletal dysfunctions through a dynamic, systems-oriented approach.

## Figures and Tables

**Table 1:** Key differences between static and dynamic networks.

Feature	Static Networks	Dynamic Networks
Node and edge change	Fixed over time	Evolving over time
Best use case	Structural/topological analysis	Temporal evolution and interactions
Example	Anatomical relationships	Neuromuscular adaptations

**Table 2:** Quantitative BIOMECH 3.4 network properties. The clustering coefficient of 0.341 suggests the presence of localized anatomical clusters supporting regional coordination. A path length of 6.43 reflects efficient biomechanical signal propagation across the system, while the low centralization and high heterogeneity indicate resilience to localized dysfunction.

Metric	Value	Interpretation
Characteristic path length	6.43	Efficient transmission of biomechanical tension
Clustering coefficient	0.341	Presence of local functional clusters (e.g., muscle groups, fascial systems)
Network density	0.003	Selective and functionally relevant connections
Diameter	14	Longest path between two nodes
Radius	7	Shortest maximum distance to all other nodes
Network centralization	0.036	Highly decentralized structure → high resilience to localized perturbations
Heterogeneity	1.332	Presence of key anatomical hubs
Connected components	1	The entire system is globally integrated

**Table 3:** Comparative table of the three main centrality metrics (betweenness, degree, and closeness centrality) analyzed using the aNETomy model and applied to the osteo-myofascial network. These nodes play a crucial role at both the local and systemic level in maintaining biomechanical balance and functional resilience.

Degree Centrality	Betweenness Centrality	Closeness Centrality
Right Humerus	Pectoralis Major—Right	Gluteus Maximus—Right
Left Humerus	Pectoralis Major—Left	Gluteal Aponeurosis—Left
Right Crural Fascia	L2	Gluteal Aponeurosis—Right
Left Crural Fascia	Spinal Dura Mater	Iliac Fascia—Left
Right Scapula	L4	Iliac Fascia—Right
Left Scapula	L3	Semispinalis Dorsi—Left
Left Tibia	Right Femur	Semispinalis Dorsi—Right
Right Tibia	Left Femur	Deep Cervical Fascia
Palmar Aponeurosis—Left	Gluteus Maximus—Left	Right Scapula
Palmar Aponeurosis—Right	Gluteus Maximus—Right	Left Scapula
Right Ilium	D7	Spinal Rotator Muscles (Dorsal)—Left
Left Ilium	Occiput	Thoracic Interspinales—Right
Tarsometatarsal Joint Capsule—Right	Dorsal Foot Fascia—Right	Thoracic Interspinales—Left
Tarsometatarsal Joint Capsule—Left	Dorsal Foot Fascia—Left	Spinal Rotator Muscles (Dorsal)—Right
Right Longissimus Dorsi	D8	Psoas Major—Right
Left Longissimus Dorsi	Left Sartorius	Psoas Major—Left
Left Antebrachial Fascia	Right Sartorius	Intervertebral Disc D12–L1
Right Antebrachial Fascia	D9	Intervertebral Disc D11–D12
Right Temporal	D10	Middle Cervical Fascia—Right
Left Temporal	Mandible	Middle Cervical Fascia—Left
Left Fascia Lata	Extensor Digitorum—Right	Right Ilium
Right Fascia Lata	Extensor Digitorum—Left	Left Ilium
Left Ulna	Gluteal Aponeurosis—Left	Renal Fascia—Left
Right Ulna	Gluteal Aponeurosis—Right	Renal Fascia—Right
Left Plantar Aponeurosis	L5	Psoas Minor—Right
Right Plantar Aponeurosis	D11	Psoas Minor—Left
Pubis—Left	Right Calcaneus	Pectoralis Major—Right
Pubis—Right	Left Calcaneus	Pectoralis Major—Left
Thoracic Fascia—Left	Nuchal Fascia—Left	2nd Rib—Right
Thoracic Fascia—Right	Nuchal Fascia—Right	2nd Rib—Left
Hyoid Bone	D6	Sacrotuberous Ligament—Left
Superficial Cervical Fascia—Left	Epicranial Aponeurosis	Sacrotuberous Ligament—Right
Superficial Cervical Fascia—Right	Iliocostalis Lumborum—Right	Intervertebral Disc L1–L2
Right Fibula	Iliocostalis Lumborum—Left	Intervertebral Disc L2–L3
Left Fibula	Frontal Bone	Occiput
Dorsal Hand Fascia—Right	D1	Intervertebral Disc L3–L4
Dorsal Hand Fascia—Left	Rectus Abdominis—Right	Intervertebral Disc L4–L5
Frontal Bone	Rectus Abdominis—Left	4th Rib—Left
3rd Metatarsal—Left	Flexor Carpi Ulnaris—Left	4th Rib—Right
3rd Metatarsal—Right	Flexor Carpi Ulnaris—Right	3rd Rib—Left
10th Rib—Left	Cranial Dura Mater—Left	3rd Rib—Right
10th Rib—Right	Cranial Dura Mater—Right	Thoracolumbar Fascia (Middle Lamina)—Right
Right Calcaneus	D3	Thoracolumbar Fascia (Middle Lamina)—Left
Left Calcaneus	D2	Serratus Posterior Inferior—Left
Middle Cervical Fascia—Right	Right Trapezius Muscle	Serratus Posterior Inferior—Right
Middle Cervical Fascia—Left	Left Trapezius Muscle	Intervertebral Disc L5–S1
2nd Rib—Right	Right Longissimus Dorsi	Supraspinous Ligament D5–D6
2nd Rib—Left	Left Longissimus Dorsi	Supraspinous Ligament D12–L1
2nd Metatarsal—Right	Right Fibula	Supraspinous Ligament D6–D7
2nd Metatarsal—Left	Left Fibula	Supraspinous Ligament D11–D12
4th Metatarsal—Left	Left Deltoid Fascia	7th Costal Cartilage—Right
4th Metatarsal—Right	Right Deltoid Fascia	7th Costal Cartilage—Left
9th Rib—Left	Flexor Retinaculum—Left	Supraspinous Ligament D7–D8
9th Rib—Right	Flexor Retinaculum—Right	Supraspinous Ligament D8–D9
11th Rib—Left	Middle Cervical Fascia—Right	Supraspinous Ligament D9–D10
11th Rib—Right	Middle Cervical Fascia—Left	Supraspinous Ligament D10–D11
12th Rib—Right	Left Ulna	D3
12th Rib—Left	Right Ulna	D1
Flexor Retinaculum—Left	Right Temporal	D2
Flexor Retinaculum—Right	Left Temporal	D5

**Table 4:** Comparative summary of centrality metrics in the aNETomy model. The potential therapeutic relevance of targeting central nodes to restore force transmission and systemic balance is highlighted.

Centrality Type	Function	Example Structures	Clinical Applications
Degree Centrality	Local force distribution	Sternum, sacrum, vertebrae	Mobility, decompression, regional integration
Betweenness Centrality	Bridge between modules	Humerus, fascia lata	Postural balance, load transfer, movement continuity
Closeness Centrality	Global coordination	Lumbar spine, sacroiliac joint	Systemic tension control, motor synchronization

## Data Availability

Data are available upon request.
